# Field evaluation of Standard Q Filariasis Antigen Test for Lymphatic Filariasis (LF) during a pre-transmission assessment survey in Sierra Leone, 2022

**DOI:** 10.1371/journal.pntd.0012773

**Published:** 2025-07-28

**Authors:** Benoit Dembele, Mohamed Salieu Bah, Abdulai Conteh, Habib I. Kamara, Ibrahim Kargbo-Labour, Ashley Souza, Patricia Houck, Ernest O. Mensah, Victoria Turay, Eric Scott Elder, Katherine Gass, Steven D. Reid, Joseph P. Shott, Yaobi Zhang, Kimberly Y. Won, Angela Weaver

**Affiliations:** 1 Helen Keller International, Regional Office for Africa, Dakar, Senegal; 2 Helen Keller International, Freetown, Sierra Leone; 3 Neglected Tropical Disease Program, Ministry of Health and Sanitation, Freetown, Sierra Leone; 4 Task Force for Global Health, NTD Support Center, Atlanta, Georgia, United States of America; 5 Helen Keller International, New York, New York, United States of America; 6 FHI 360, USAID’s Act to End Neglected Tropical Diseases (NTDs) West Program, Abidjan, Cote d’Ivoire; 7 Centers for Disease Control and Prevention, National Center for Emerging and Zoonotic Infectious Diseases, Division of Parasitic Diseases and Malaria, Atlanta, Georgia, United States of America; 8 Division of Neglected Tropical Diseases Office of Infectious Diseases, Bureau for Global Health USAID, Washington, DC, United States of America; The University of Melbourne, AUSTRALIA

## Abstract

**Background:**

As part of a multi-country evaluation, the SD Biosensor STANDARD Q Filariasis Antigen Test (QFAT) was compared with the Abbott Bioline Filariasis Test Strip (FTS) for assessing *Wuchereria bancrofti* infection prevalence and for ease of use of QFAT in field conditions in Sierra Leone.

**Methods and principal findings:**

The evaluation was done in two districts, Bombali and Karene, where repeat pre-transmission assessment surveys (pre-TAS) were planned. Two rural sites, Kagbo and Makorba Yelimini which recorded high LF antigen prevalence of 4.1% and 7.7% respectively in repeat pre-TAS in 2020 were selected for the QFAT evaluation. At least 350 people ≥5 years were targeted per site for inclusion in the study by convenience sampling. Blood was collected by fingerstick; 20μl for QFAT and 75μl for FTS. The reading time for both tests was 10 minutes. For all positive or invalid results, a repeat test was performed for both tests. In total, 728 participants (5–91 years) were tested by QFAT and FTS. Four FTS and three QFAT final results were classified as indeterminate (meaning a positive result followed by a negative result) and were excluded from the analysis. The positive rate was 4.8% (17/357) and 3.5% (13/367) for FTS and 3.4% (12/359) and 4.1% (15/366) for QFAT in Kagbo and Makorba Yelimi, respectively. All participants testing positive for FTS or QFAT underwent further testing by night blood smear to detect microfilariae using microscopy. No *W. bancrofti* microfilariae was detected in any of the antigen positive cases. In field conditions, QFAT was easy to handle and recorded zero invalid tests compared to FTS (six invalids). The concordance between FTS and QFAT was 0.81 (Cohen’s Kappa). The discrepancy found between the two tests in terms of positive tests was not statistically significant (p = 0.78).

**Conclusions/significance:**

The results suggest that the QFAT is a credible *W. bancrofti* diagnostic test when compared to the routinely used FTS; use of either test would result in the same program decision.

## Author summary

Lymphatic filariasis (LF) is a vector-borne disease targeted for elimination as a public health problem by 2030. The Global Program to Eliminate LF recommends tools to measure the impact of interventions on infection prevalence and when elimination has been achieved. A reliable and easy to use diagnostic tool is key for the success of the global program. Currently only one rapid antigen test is used for programs in *Wuchereria bancrofti* endemic countries. This study was part of a multi-country field evaluation of a new *W. bancrofti* antigen lateral flow assay rapid diagnostic test, the SD Biosensor STANDARDQ Filarial Antigen Test (QFAT). The primary objective was to determine comparability of QFAT to the Abbott Bioline Filariasis Test Strip (FTS) in its ability to classify LF prevalence at a population level and compare performance of QFAT to FTS. In addition, information was collected on the ease of use of QFAT in field settings. The evaluation was done in two districts (Bombali and Karene) in Serra Leone, which were undergoing repeat pre-transmission assessment surveys (pre-TAS). The results of the study confirm QFAT as a suitable alternative to the currently recommended FTS. In field conditions, using QFAT seems effective given that no invalid test was recorded compared to six invalid FTS tests.

## Introduction

Lymphatic filariasis (LF) is a preventable mosquito-borne infectious disease, caused by infection with one of the filarial parasites, *Wuchereria bancrofti*, *Brugia malayi* or *Brugia timori.* The infection impairs the lymphatic system, where the worms nest, later manifesting as hydrocoele, lymphoedema or elephantiasis in some individuals, resulting in unnecessary physical and mental suffering [1].

*W. bancrofti* is found in nearly all LF endemic countries whereas *Brugia spp*. are primarily reported in Southeast Asia but have also been documented in parts of South Asia, including Sri Lanka [2]. *W. bancrofti* is responsible for 90% of LF infections [3]. The World Health Organization (WHO) established the Global Program to Eliminate Lymphatic Filariasis (GPELF) to eliminate LF by mass drug administration (MDA) of anthelminthics and to alleviate the suffering of people affected by the disease through morbidity management and disability prevention (MMDP). The current target is to achieve elimination in 81% of endemic countries by 2030 [4]. By 2022, 19 of the 72 endemic countries had successfully eliminated LF as a public health problem. However, there are still an estimated 794 million people requiring MDA worldwide [1]. To achieve LF elimination, a country follows four recommended sequential programmatic steps: baseline mapping, MDA implementation, post-MDA surveillance, and validation of LF elimination. Transition from one stage to the next is dependent on the availability of user-friendly and highly sensitive and specific rapid diagnostic tools [5]. Within GPELF, Transmission Assessment Surveys (TAS) are conducted after multiple rounds of mass drug administration (MDA) to determine whether infection levels have been reduced below the transmission threshold, thereby assessing whether MDA can be stopped [6]. Typically, an antigen (Ag) positivity rate below 2% in sentinel and spot-check sites, known as pre-transmission assessment surveys (pre-TAS), followed by LF TAS with Ag prevalence below 2% in a broader evaluation area, indicates that transmission is unlikely to be sustained [6]. Accurate diagnostic tools are essential at this stage to ensure that low-level infections are not missed, which could lead to recrudescence in endemic areas [7].

Previously, Sierra Leone was endemic for LF throughout the country [8]. LF MDA started in Sierra Leone in 2007, achieving 100% geographical coverage in 2009 [9,10]. Despite various challenges, the country has achieved great progress toward LF elimination [9,11,12]. To date, 12 of 16 districts have stopped LF MDA and transitioned to post-MDA surveillance [6]. Four districts (Bombali, Karene, Koinadugu, and Falaba) remained under MDA. The four districts failed a third pre-TAS in 2020 and have since conducted two annual rounds of MDA with ivermectin and albendazole in 2021 and 2022. The districts were scheduled to conduct a fourth repeat-pre-TAS using FTS in 2022 six months after the last MDA in line with WHO recommendations [6,13]. The districts would be eligible for TAS if all pre-TAS sites had less than 2% LF antigen prevalence. The inclusion of circulating filarial antigen (CFA) detection in the survey provided a good opportunity for a comparative evaluation of QFAT and FTS.

Where *W. bancrofti* is the causative agent of LF, the Alere Filariasis Test Strip (FTS, Abbott) is currently the main diagnostic tool recommended for program use [14]. The FTS detects CFA of the adult worm. However, FTS has limitations. First, it requires sufficient time to collect 75 microliters (μL) of blood using micropipettes, which increases the risk of clotting, preventing proper flow of the blood sample on the test strip. Second, FTS consists of a single lightweight nitrocellulose strip devoid of any protective housing (e.g., a plastic cassette) and a lightweight plastic container, which has created logistical challenges, requiring survey teams to secure the test strips with tape to minimize movement during testing.

A new rapid diagnostic test, the STANDARD Q Filariasis Antigenemia Test (QFAT) (SD Biosensor, Suwon, South Korea) also detects CFA of *W. bancrofti* and may be an alternative tool for LF programs. The QFAT cassette form design, with less blood sample (20 μL) required, and inclusion of a buffer solution to aid in sample flow represent potential improvements compared to the FTS. A multi-country field evaluation of the QFAT was planned including this study in Sierra Leone in Africa in others in the Asia-Pacific regions [15–17]. Here, we report comparison of the QFAT to FTS in Sierra Leone. The purpose of the study was to compare the performance of QFAT in detecting CFA to FTS in field setting in two LF endemic districts in Sierra Leone. The primary objectives were: 1) to determine the comparability of the QFAT to the FTS in its ability to classify LF prevalence at a population level; 2) compare performance of QFAT to FTS and 3) to understand the end-user experience with the new QFAT.

## Methods

### Ethical considerations

The Ethics and Scientific Committee, Ministry of Health and Sanitation, Sierra Leone approved the survey protocol (SLESRC No:010/06/2022). Consent documents and participation information were provided and explained to participants by team members who were fluent in the appropriate local language. Adult participants provided written informed consent, while parents or guardians provided written consent for minors (5 – 17 years). The community leaders were informed about the survey and they granted permission to conduct the survey in their respective communities. To ensure privacy and confidentiality, an identification barcode was assigned to each participant and used only for sample tracking. Personal identifying information was kept confidential. All confirmed positive and indeterminate cases were treated with ivermectin and albendazole by the survey team based on the survey protocol.

### Study sites

The study was conducted in the context of re-pre-TAS in two villages in Sierra Leone: Kagbo in the Safroko Limba chiefdom in Bombali district and Makorba Yelimi in the Sanda Loko chiefdom in Karene district from July 18– 22, 2022. Bombali was redistricted in 2020 to create two districts, Bombali and Karene. MDA with co-administration of ivermectin and albendazole started in Bombali in 2008. After five rounds of MDA with epidemiological coverage > 65%, the first pre-TAS was conducted in 2013. The district failed to meet the established pre-TAS threshold for continuing to TAS. The district failed two more pre-TAS in 2017 and 2020. In each case, at least two additional annual MDA rounds with epidemiological coverage > 65% were carried out before a repeat pre-TAS was conducted. The most recent MDA in Bombali and Karene was conducted in December 2021, more than six months prior to the fourth re-pre-TAS. Makorba Yelimi was a spot check site in the 2017 and 2020 pre-TAS and had antigen prevalence of 25.9% and 7.7%, respectively. In the 2020 pre-TAS Kagbo had an antigen prevalence of 4.1%. In all surveys, FTS was used to assess *W. bancrofti* antigenemia prevalence. In the 2022 re-pre-TAS, the two districts were assessed separately as two evaluation units. Bombali and Karene were selected for this study because antigen-positive cases were likely to be found in the two districts, facilitating the comparison between the QFAT and FTS. Kagbo and Markoba Yelimi were selected from six available sites since both were remote rural communities, hard-to-reach, and had a history of high parasite prevalence.

### Sample size

The target sample size for the study was 538 individuals across the two sites. This was calculated using McNemar’s paired test for binary outcomes, with a non-inferiority margin of 2%, an expected prevalence of 5%, 75% power and 5% statistical significance. To ensure the sample size was met, survey teams were encouraged to collect samples from at least 350 individuals per site, which also aligns with WHO’s minimum sample size requirements for a pre-TAS survey.

### Participant enrollment

During the survey, the team arrived at each community early to brief the stakeholders and set-up enrollment and data collection points in community designated sites. Participants were enrolled by convenience sampling, the standard sampling method for pre-TAS. Community members were invited to participate through announcements by community leaders, community drug distributors and health workers from the nearest health center visiting each home. People > 5 years were enrolled as they presented to the survey point and consented to participate. Demographic data (name, age, sex) were collected. Each participant was assigned a unique identification number using a QR code, which was used to label the consent form, samples collected, and the tests for the same person.

### Sample collection

Before the survey, two tests from each batch of FTS were tested with a positive control sample obtained from WHO at the Central Public Health Laboratory in Freetown. For the QFAT, quality control testing using the same positive control sample was carried out during the survey team’s training. All test kits were stored at ambient temperature (16°-24°C) in the laboratory before the survey.

Sample collection protocols differed between the two study sites. However, all tests were conducted immediately after specimen collection. In Kagbo village, two technicians were involved in blood collection and conducting the tests. First, the technicians pricked the finger of a participant using a sterile disposable lancet. A 20µl blood sample was collected directly from the finger prick using the sample collection device provided with the QFAT kit. Following manufacturer’s instruction, the blood sample was placed on the cassette sample port, and two drops of buffer solution were added. Then, approximately 190µl of finger prick blood was collected into a heparinized microtainer tube (Ram Scientific). Subsequently, 75µl of blood was removed from the heparin tube using a calibrated pipette and added slowly to the lower half of the sample pad of the FTS. QFAT and FTS results were read at exactly 10 minutes (recorded by digital timers) according to manufacturer instructions. In Makoba Yelimi village, blood samples were directly collected from the finger prick using the manufacturer provided sample collection devices for the FTS (75µl) and QFAT (20µl) and applied to the tests immediately. Tests were performed according to manufacturer instructions.

All positive and invalid (i.e., absence of control line or failure of complete sample migration on test strip) results for FTS or QFAT were repeated immediately using remaining blood in heparin tubes in Kagbo or a fresh blood sample in Makoba Yelimi.

Following WHO recommendations, individuals who were confirmed positive by FTS or QFAT were followed up for a night blood specimen collection during the hours of peak microfilariae (Mf) circulation (10 pm to 2 am). A blood sample was collected directly from the finger prick and gently placed on a slide as three lines along the length of the slide giving a total of 60μl of finger prick blood for each slide (3x20µl). Two slides were prepared per individual. The slides were transported to the Neglected Tropical Disease laboratory in Makeni where they were stained with Giemsa and examined for the presence of Mf.

### Ease of use questionnaire

Standardized questionnaires were used to collect the technicians’ opinions on the ease-of-use of QFAT. All the field technicians were familiar with using FTS having used it in more than three previous pre-TAS or TAS. A self-administered electronic questionnaire was sent to the five technicians who implemented the tests to collect their opinions on ease of use of QFAT (questionnaire in S1 Information). The technicians graded (strongly disagree, disagree, agree or strongly agree) questions related to QFAT instructions for use, kit packaging and labeling, kit packing material, test procedures, and reading and interpretation of results.

All data were entered into an electronic form using Open Data Kit (ODK) and Ona platform.

### Data analysis

Data from the ODK website were exported into Microsoft Excel, cleaned and analyzed using Statistical Package for Social Science (SPSS version 20) and Epi info 7. The primary outcome was assessed using McNemar’s chi-squared test to compare the antigen prevalence between the two tests, with a p-value < 0.05 considered as evidence of a statistically significant difference between the two prevalences. The chi-square test was also used to compare the Ag prevalence by age group, location, and sex. To assess whether the tests’ performance differed according to demographic group, we analyzed results according to sex and age group (5–9, 10–14, 15–19, ≥ 20 years). To assess for any geographical differences in performance, the site level prevalence by FTS or QFAT, estimated as the number of positive cases out of the number of valid tests, was compared along with the 95% confidence interval (CI). Cohen’s Kappa coefficient was assessed to further investigate the agreement between the two tests. We interpreted the Cohen’s Kappa coefficient as follows: < 0.00 poor agreement, 0.00-0.20 slight agreement, 0.21-0.40 fair agreement, 0.41-0.60 moderate agreement, 0.61-0.80 substantial agreement and then 0.81-1.00 almost perfect agreement. Finally, the results of the ease-of-use survey, using a Likert scale, were compiled into a table.

## Results

### Characteristics of participants and test results

In total, 728 participants aged 5–91 years were tested by both QFAT and FTS from the two sites. Of the participants with valid tests, 380 (52.5%) for FTS and 382 (52.7%) for QFAT were female. There were six invalid tests for FTS, but none for QFAT. Five FTS-invalid tests occurred in the first tests, while one occurred in the repeat of a positive test. Two of the six FTS-invalid tests were QFAT positive. The proportion of the participants by age group is shown in Table 1. Four FTS and three QFAT indeterminate results (Table 2) were excluded from the analysis.

**Table 1:** FTS and QFAT result distribution by age group, sex and sites.

Group	No of persons with valid FTS test	No of persons testing FTS positive	Positive rate (%) by FTS (95% CI)	No of persons with valid QFAT test	No of persons testing QFAT positive	Positive rate (%) by QFAT (95% CI)
Total	724	30	4.14 (2.92-5.85)	725	27	3.72 (2.57-5.36)
By age (in years)						
5-9	127	0	0	128	0	0
10-14	172	2	1.16 (0.14-4.14)	172	2	1.16 (0.14-4.14)
15-19	81	1	1.23 (0.03-6.69)	81	1	1.23 (0.03-6.69)
≥20	344	27	7.85 (5.45-11.18)	344	24	6.98 (4.73-10.1)
By sex						
Male	344	16	4.65 (2.88-7.42)	343	15	4.37 (2.67-7.09)
Female	380	14	3.68 (2.21-6.09)	382	12	3.14 (1.81-5.41)
By village						
Kagbo	357	17	4.76 (2.99-7.49)	359	12	3.34 (1.92-5.75)
Makorba Yelimi	367	13	3.54 (2.08-5.97)	366	15	4.10 (2.50-6.65)

Concordance between QFAT and FTS.

**Table 2:** Concordance of results between QFAT and FTS.

	FTS positive	FTS negative	FTS Indeterminate	Total
QFAT positive	22	4	1	27
QFAT negative	6	689	3	698
QFAT Indeterminate	2	1	0	3
Total	30	694	4	728

The concordance test between FTS and QFAT, Cohen’s Kappa was 0.81 indicating almost perfect agreement between tests.

### Prevalence of *W. bancrofti* and performance of QFAT against FTS

In terms of the primary study objective, of the 724 and 725 participants with valid test results for FTS and QFAT, respectively, 4.14% (95% CI: 2.92-5.85%) were antigen positive by FTS, whereas 3.72% (95% CI: 2.57-5.36%) were positive by QFAT. There was no statistically significant difference between the results of the two tests (χ^2^ = 0.10, p = 0.75).

A further analysis of the positivity rates at the village level found no significant difference between the two tests in Kagbo (χ^2^ = 0.60, p = 0.43) or Makorba Yelimi (χ^2^ = 0.04, p = 0.84). None of the positive participants had circulating Mf from the midnight blood tests. The greatest proportion of people testing positive was in the oldest age group, regardless of the type of test kit used, with 7.85% of participants ≥20 years positive by FTS and 6.98% positive by QFAT. No positive case was found in the age group of 5–9 years old for either test. More males than females were positive by both tests; however, there was no statistical difference between the two tests when comparing FTS vs. QFAT among females (χ^2^ = 0.03 p = 0.86) nor among male (χ^2^ = 0.17 p = 0.68).

### User experience of QFAT

The five team members who used the QFAT responded to the user experience questions. All test users generally agreed on their responses to the ease-of-use questionnaire. The questions were related to instructions for use, kit packaging and labelling, kit packaging material, device assay procedure, interpretation of result (Table 3). All the answers from the technicians except one were either agree or strongly agree with more strongly agree responses than agree.

**Table 3:**
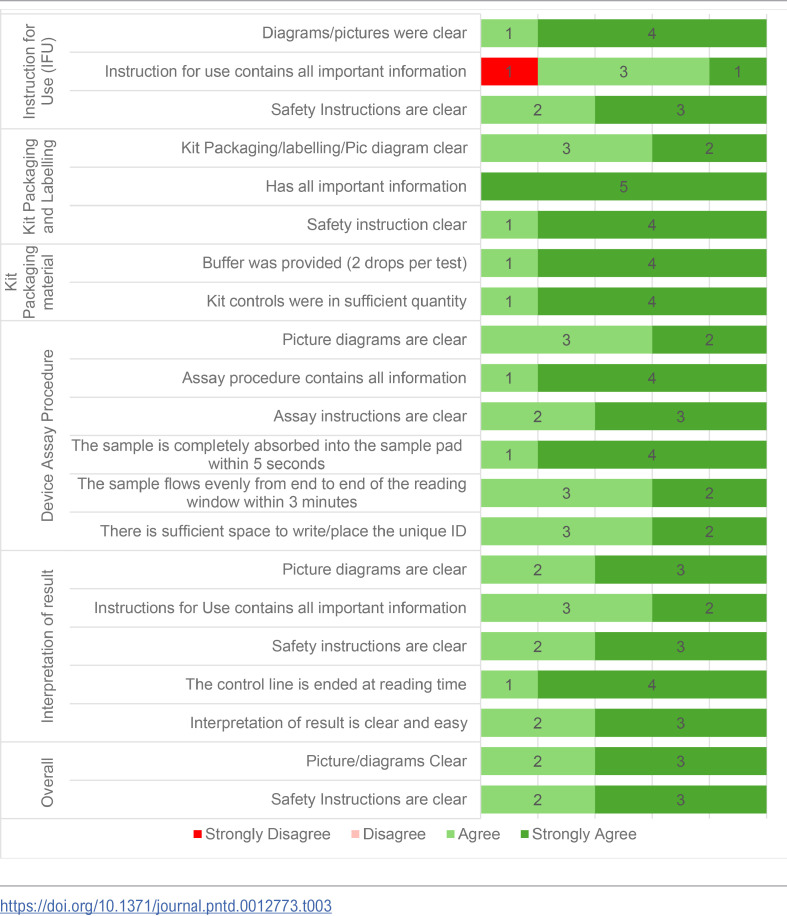
QFAT user survey summary.

## Discussion

This study, conducted in two villages in Sierra Leone, was the first field application of the new QFAT to detect LF antigenemia in Africa. The main purpose of the study was to compare the performance of the QFAT to the FTS in terms of its ability to detect LF antigenemia at a population level. The QFAT was found to have comparable performance to the FTS; there was no statistically significant difference between the tests. Furthermore, both villages recorded filarial antigen prevalence of >2% by either FTS or QFAT test, indicating that the same program decision (failing to pass the re-pre-TAS) was made based on the results of either test. The prevalence of LF Ag was comparable by sex for both tests. The Cohen’s Kappa coefficient, a measure of agreement between the two tests, was 0.81, suggesting an almost perfect agreement. These metrics indicate that the QFAT performs reliably as a valid diagnostic tool for LF in field conditions, as compared to the performance of the FTS. This result corroborates that of the QFAT/FTS comparison study carried out in a field laboratory in Samoa, where the concordance rate was 0.85 [15]. Laboratory comparisons of QFAT and FTS using serum or plasma samples showed that the QFAT was a suitable rapid antigen test for use in LF elimination programs and had advantages over FTS in ease-of-use, smaller sample, clearer control line, and higher sensitivity for Mf-positive samples [16].

While no significant difference in prevalence was observed between the two tests at either village site, in Makorba Yelimi, where the blood sample collection device provided with the FTS kit was used to collect blood and then apply to the FTS, we found fewer FTS positives compared to QFAT. The reverse was the case in the first site where blood samples were first collected into heparin tubes followed immediately by pipetting 75µl for the FTS test. The reasons for the difference are unclear besides the fact that using the pipette may have ensured that adequate blood was used for FTS testing, which may not be the case when using the sample collection device, likely due to user inconsistency in obtaining adequate blood sample each time.

One of the primary goals of the study was to assess the feasibility and user-experience of the QFAT in field conditions. The fact that the quantity of blood required for the QFAT is smaller (20 µl) than for the FTS (75 µl) makes it less challenging for surveyors in the field to collect a sufficient volume of blood for the test. Although only a relatively small number of team members completed the ease-of-use questionnaire, responses indicated generally strong agreement that the QFAT kit included good instructional materials and was well packaged. In addition, the QFAT test cassettes can easily be labelled with participant identification and test results, unlike FTS, which need to be fixed to the test strip reservoir [18]. Assessing user experience in a standardized way provided insight on how the QFAT performed in real-world conditions and confirmed there were no significant barriers to its use. While the QFAT requires an additional step of adding a buffer solution that is not required with the FTS, the inclusion of the buffer solution facilitated the migration of the blood sample and high throughput, while poor migration is often observed with the FTS a possible cause of invalid FTS. The sample collection device for QFAT was easy and more convenient for blood collection than the FTS device. The difference in shelf life (12 months for FTS vs. 24 months for QFAT) has important implications for program use.

There are some limitations in our study. The QFAT was added to the scheduled re-pre-TAS design, therefore the number of samples to be tested was limited by the WHO recommended pre-TAS sample size. The fact that all the mf results were negative was not helpful to compare the sensitivity of the two tests. However, some other studies made that comparison for the QFAT, and both tests were positive for all the Mf-positives samples [15]. Those limitations did not impact the fact that the study reached the primary goal to compare the performance of the FTS and QFAT in field conditions. While we did not directly assess the test line intensity in our qualitative evaluations, the end users noted that the QFAT test line was sometimes faint and thin, possibly making it challenging to interpret positive results accurately. This was one of the noted disadvantages of the QFAT in this study. This may lead to difficulties in field conditions where clarity and ease of result interpretation are crucial for timely and accurate decision-making. To improve the QFAT and address the issue of thin test lines, it is suggested that developers focus on several key areas. They could enhance line visibility by increasing the contrast and thickness of the positive test line to make results more pronounced and easier to interpret. Providing comprehensive training and clear guidelines for interpreting test results is also crucial. Lastly, implementing robust quality assurance programs is essential to ensure that test kits meet high standards and perform consistently across different settings. Quality assurance programs, as highlighted by Gass et al. (2012) [7], are critical for maintaining the reliability of diagnostic tests in field conditions. These combined efforts may enhance the reliability of the QFAT, making it an effective tool for diagnosing LF.

## Conclusions

This study provided the first field-based evaluation of the new QFAT, demonstrating its comparable performance to the widely used FTS. By showing almost perfect agreement between the two tests and consistent programmatic outcomes, the study outcomes supports QFAT as a reliable diagnostic test for the detection of *W. bancrofti* CFA and therefore a useful diagnostic tool for LF elimination programs. Its ease of use, high throughput, and zero invalid test results further enhance its practicality. However, more investigations are required in different prevalence settings to confirm that the QFAT is a reliable alternative for the FTS. Overall, this field evaluation of the QFAT in a program setting demonstrated comparable performance to the FTS and greater ease of use than FTS in field conditions. Our results suggest that QFAT can be used in GPELF as an alternative to the FTS.

## Supporting information

S1 Information Ease of use of the questionnaire of the QFAT. (DOCX)

S2 Information Study database. (XLSX)
