# Supplementary material for: Field evaluation of Standard Q Filariasis Antigen Test for Lymphatic Filariasis (LF) during a pre-transmission assessment survey in Sierra Leone, 2022
Source: PLoS Negl Trop Dis. 2025 Jul 28;19(7):e0012773. doi: 10.1371/journal.pntd.0012773 (PMC12352871; doi:10.1371/journal.pntd.0012773)
Supplement: S1 Information — (DOCX) [file pntd.0012773.s001.docx]

**FORM 3. EASE OF USE**

|  |  | **Not applicable** | **Strongly disagree** | **Disagree** | **Agree** | **Strongly agree** |
| --- | --- | --- | --- | --- | --- | --- |
| **1** | **Instruction for use (IFU)** | | | | | |
| 1.1 | Pictures/diagrams are clear |  |  |  |  |  |
| 1.2 | The IFU contains all important information |  |  |  |  |  |
| 1.3 | Safety instructions are clear |  |  |  |  |  |
| **2** | **Kit packaging and labeling** | | | | | |
| 2.1 | Kit Packaging/labelling/Pic diagram clear |  |  |  |  |  |
| 2.2 | Has all important information |  |  |  |  |  |
| 2.3 | Safety instruction clear |  |  |  |  |  |
| **3** | **Kit Packaging and material** | | | | | |
| 3.1 | Kit packaging material does not have any visible defects |  |  |  |  |  |
| 3.2 | Buffer was provided in sufficient quantity to use as directed (2 drops per test) |  |  |  |  |  |
| 3.3 | Kit controls were provided in sufficient quantities |  |  |  |  |  |
| **4** | **Device and assay procedure** | | | | | |
| 4.1 | Picture diagrams are clear |  |  |  |  |  |
| 4.2 | Assay procedure contains all information |  |  |  |  |  |
| 4.3 | Assay instructions are clear |  |  |  |  |  |
| 4.4 | The sample is completely absorbed into the sample pad within 5 seconds |  |  |  |  |  |
| 4.5 | The sample flows evenly from end to end of the reading window within 3 minutes |  |  |  |  |  |
| 4.6 | There is sufficient space to write/place the unique ID |  |  |  |  |  |
| **5** | **Reading and interpretation** | | | | | |
| 5.1 | The test line is always clear at the recommended reading time |  |  |  |  |  |
| 5.2 | The test line is always easy to read at the recommended reading time |  |  |  |  |  |
| 5.3 | The control line is always visible at the recommended reading time |  |  |  |  |  |
| 5.4 | The control line is always easy to read at the recommended reading time |  |  |  |  |  |
| 5.5 | Interpretation of the test is clear and easy |  |  |  |  |  |
| **6** | **Overall appraisal** | | | | | |
| 6.1 | Pic/diagrams Clear |  |  |  |  |  |
| 6.2 | Safety Instructions are clear |  |  |  |  |  |

If disagree or strongly disagree, report item number and describe

| **n°** | **Item** | **Comment** |
| --- | --- | --- |
|  |  |  |
|  |  |  |
|  |  |  |

Additional comments:
